# Supplementary material for: Complexome profiling of the Chlamydomonas psb28 mutant reveals TEF5 as an early PSII assembly factor
Source: Plant Cell. 2025 Mar 17;37(6):koaf055. doi: 10.1093/plcell/koaf055 (PMC12164590; doi:10.1093/plcell/koaf055)
Supplement: koaf055_Supplementary_Data [file koaf055_supplementary_data.zip › psb28_interactive.zip!/plots/intensity/qualityPlots/peptides/PGL1_Cre01.g034400.t1.1.html]

### Cre01.g034400.t1.1

Trivial name: PGL1  
SeqCoverage [median]: 11.10  
Mass: 38.94 kDa  
  
 

| Sequence | MissedCleavage | Mass [Da] | wtIntensity | muIntensity | Score |
| --- | --- | --- | --- | --- | --- |
| IISIPAAALPR | 0 | 1120.70 | 624.59 | 1203.05 | 70.92 |
| LVEEAAAEAVK | 0 | 1128.60 | 0.00 | 355.83 | 66.62 |
|  |  |  | 624.6 | 1558.9 |  |
